# Single Molecule Analysis of Replicated DNA Reveals the Usage of Multiple KSHV Genome Regions for Latent Replication

**DOI:** 10.1371/journal.ppat.1002365

**Published:** 2011-11-03

**Authors:** Subhash C. Verma, Jie Lu, Qiliang Cai, Settapong Kosiyatrakul, Maria E. McDowell, Carl L. Schildkraut, Erle S. Robertson

**Affiliations:** 1 Department of Microbiology & Immunology, University of Nevada, Reno, School of Medicine, Center for Molecular Medicine, Reno, Nevada, United States of America; 2 Department of Microbiology and Tumor Virology Program of the Abramson Cancer Center, Perelman School of Medicine at the University of Pennsylvania, Philadelphia, Pennsylvania, United States of America; 3 Department of Cell Biology, Albert Einstein College of Medicine, Bronx, New York, United States of America; Rosalind Franklin University of Medicine and Science, United States of America

## Abstract

Kaposi's sarcoma associated herpesvirus (KSHV), an etiologic agent of Kaposi's sarcoma, Body Cavity Based Lymphoma and Multicentric Castleman's Disease, establishes lifelong latency in infected cells. The KSHV genome tethers to the host chromosome with the help of a latency associated nuclear antigen (LANA). Additionally, LANA supports replication of the latent origins within the terminal repeats by recruiting cellular factors. Our previous studies identified and characterized another latent origin, which supported the replication of plasmids ex-vivo without LANA expression in *trans*. Therefore identification of an additional origin site prompted us to analyze the entire KSHV genome for replication initiation sites using single molecule analysis of replicated DNA (SMARD). Our results showed that replication of DNA can initiate throughout the KSHV genome and the usage of these regions is not conserved in two different KSHV strains investigated. SMARD also showed that the utilization of multiple replication initiation sites occurs across large regions of the genome rather than a specified sequence. The replication origin of the terminal repeats showed only a slight preference for their usage indicating that LANA dependent *origin* at the terminal repeats (TR) plays only a limited role in genome duplication. Furthermore, we performed chromatin immunoprecipitation for ORC2 and MCM3, which are part of the pre-replication initiation complex to determine the genomic sites where these proteins accumulate, to provide further characterization of potential replication initiation sites on the KSHV genome. The ChIP data confirmed accumulation of these pre-RC proteins at multiple genomic sites in a cell cycle dependent manner. Our data also show that both the frequency and the sites of replication initiation vary within the two KSHV genomes studied here, suggesting that initiation of replication is likely to be affected by the genomic context rather than the DNA sequences.

## Author Summary

Kaposi's sarcoma associated herpesvirus (KSHV) establishes lifelong infection in the infected host and induces lymphoproliferative diseases, body cavity based lymphomas and sarcomas in immune compromised individuals. Herpesviruses including KSHV uses host cellular replication machinery for the replication of their genome. Here, for the first time we show that KSHV not only uses the host cellular machinery for its replication but also uses a similar mechanism for replication initiation at replication zones. KSHV was able to initiate replication throughout the genome thus the entire genome may act as a replication initiation zones. These data propose that replication initiations are not determined by the specificity of sequences but the genetic context of the genome and so suggest that epigenetic modification may play an important role in initiating DNA replication. Broadly, these results shed light on the evolutionary trends of large oncogenic dsDNA virus replication, which is similar to the replication of cellular DNA and therefore provides a strategy for the viruses to escape the host immune surveillance.

## Introduction

Kaposi's sarcoma associated herpesvirus, also referred to as human herpesvirus 8 (HHV8), belongs to the gammaherpesvirus family and is associated with multiple lymphoproliferative diseases including Body Cavity Based Lymphomas (BCBLs) and Multicentric Castleman's Disease (MCDs) [1], [2], [3]. KSHV, like other herpesviruses establishes lifelong infection in the infected hosts and maintains the viral genome as extra-chromosomal episomes in a latent state [4], [5], [6]. The virus encodes a limited number of genes for persistence without being recognized by the host immune surveillance [7], [8], [9]. Latency Associated Nuclear Antigen (LANA) is one of the proteins expressed in all latently infected cells [5], [10], [11]. LANA is considered an oncogenic protein because of its role in modulating cellular pathways required to induce/promote tumorigenesis [12], [13], [14], [15]. LANA has also been shown to degrade the tumor suppressors, p53, pRb and von Hippel Lindau (VHL) by recruiting ubiquitin ligases [13], [16], [17], [18]. LANA has also been shown to upregulate the proteins important for immortalization of infected cells including upregulation of hTERT [12], [19]. Along with its role in modulation of various cellular and viral pathways, LANA is critical for maintaining the viral genome in infected cells [5], [6], [20]. LANA docks onto the host chromatin through the amino terminal chromatin-binding domain (CBD) and tethers the viral genome to the host chromosome by binding to the DNA binding domain of the carboxyl terminus within the terminal repeats [5], [6], [21], [22].

The KSHV genome has multiple reiterated copies of the terminal repeats (TR), which are proposed to be the region required for circularization of the genome. Each terminal repeat unit is a 801 bp long high GC content DNA element and was shown to contain the latent origin, or replication initiation site similar to EBV [23], [24], [25], [26]. Each TR unit has two LANA binding sites (high affinity site termed as LBS1 and lower affinity one termed LBS2) [24]. A 31 bp long sequence upstream of the LANA binding sequences is mapped as a replicator element (RE), which is important for replication initiation [24], [26]. Plasmids containing the RE element along with the LANA binding sequences is replication sufficient in a LANA dependent manner [26]. Comparison of the functional *replication origins* of KSHV with EBV showed that these two viruses differ in sequence homology but retain significant structural similarities [24]. For example, the terminal repeats of KSHV has two LANA binding sites, high and low affinities (LBS1 and LBS2) similar to the high and low affinity EBNA1 binding sites in the dyad symmetry element of EBV [24]. This suggests that both LANA and EBNA1 may share similar functions in terms of recruitment of cellular proteins [24]. A single copy of the TR with both the LANA binding site (LBS1/2) and RE is able to support the transient replication of a plasmid but requires at least two copies of the TR for stable episomal maintenance [23], [27]. Similar to KSHV, EBV *ori*P can also replicate with 2 copies of the EBNA1 binding site but requires an additional EBNA1 binding site within the family of repeats for stable maintenance [27], [28], [29]. Both, EBNA1 and LANA have been shown to stimulate DNA replication by directly recruiting the host cellular replication machinery [30], [31], [32], [33], [34]. Origin recognition complexes (ORCs) are the essential proteins which form the pre-replication complex (pre-RC) by recruiting the minichromosome maintenance proteins (MCMs) [35]. Both, LANA and EBNA1 have been shown to recruit ORC and MCM proteins at the replication origins and are important for latent replication [30], [32], [33], [34]. We have shown that licensing of the replication factors at these origins occurs once per cell cycle, which is similar to the cellular replication origins [26]. We have also shown that the initiation of DNA replication at TR is regulated by Geminin, which blocks replication during the same cell division [26]. Work from our lab and others clearly demonstrated that each TR unit has a replicator element [24], [26]. However, our recent work demonstrated the presence of an additional replication site at the left end of the KSHV genome [36]. This replication site does not require expression of LANA in trans, therefore we will refer to this as an autonomous replication origin (*ori*A throughout the manuscript) [36]. We identified *ori*A by screening the 33 kb left end of the genome by subcloning this cis-element into a plasmid and analyzing its ability to support replication [36]. Cellular replication proteins accumulated at the *ori*A in a cell cycle dependent manner to form *pre*-RC [36]. The presence of additional replicator sequences encouraged us to analyze the entire KSHV genome for potential replicator elements.

We used the single molecule analysis of the replicated DNA (SMARD) technology to identify the replication zones of individual KSHV episomes. SMARD is a powerful method for detecting replication initiation sites on the individual molecules using fluorescence microscopy [37]. This technology has been used for understanding the replication initiation and the dynamics of replication fork movement of Epstein Barr Virus [38]. SMARD has also been used for characterizing a number of replication origins in cellular DNA [39], [40].

In this study, we performed an extensive analysis of replication initiation sites and the replication dynamics of KSHV genome in two human body cavity based lymphoma cell lines (BCBL-1 and JSC-1). By stretching the DNA molecules extracted from latently infected cells, we were able to collect sufficient numbers of KSHV genomes representing the various stages of the replication fork movement. SMARD allowed us to determine the replication initiation site, progression of the replication fork, and termination of replication on the KSHV genome. Since this technique determines the replication fork progression in a steady state, it can also be used to calculate the duplication time needed to replicate specific regions of the KSHV genome. Our data now shows that replication initiation events can occur throughout the KSHV genome, which is distinctly different from earlier conclusions that replication initiates from a specific site within the terminal repeats. Our data also shows that both, the frequency and the sites of replication initiation vary within the two-studied KSHV genomes (BCBL-1 and JSC-1) suggesting that initiation of replication is likely affected by the genomic context rather than the DNA sequences. Detection of protein components of the pre-RC on the genome by chromatin immunoprecipitation assay shows accumulation of ORC2 and MCM3 on various regions of the KSHV genome suggesting potential replicator sites. These data suggests that replication initiates at various regions of the KSHV genome, and is primarily controlled by the genomic context rather than specific sequences.

## Results

### Latent KSHV genomes incorporated halogenated nucleotides analogs that are detected by immunostaining

In order to delineate the DNA replication initiation sites of the KSHV genome, we used BCBL-1 and JSC-1 PEL cells which maintain the latent viral genome. These two cell lines were labeled with nucleotide analogs, IdU (5′-iodo-2′-deoxyuridine) and CldU (5′-chloro-2′-deoxyuridine) for fluorescent visualization of DNA replication across the genome using a previously established technique, single molecule analysis of the replicated DNA (SMARD) [37], [38]. In this technique, the replicating DNA is labeled in a way, which helps us to determine the position, direction and the abundance of regions of replication forks on replicated molecules. This in turn allows us to determine the replication initiation sites, progression of replication forks and replication termination sites on the genome analyzed. In this labeling procedure, asynchronously growing cells are sequentially labeled with IdU and CldU for the complete duplication of the KSHV genome (4 h). This allows the replicating molecules to incorporate the halogenated nucleotides throughout the entire length of the genome [38]. These cells are switched from the first label (IdU) to the second label (CldU) after 4 h, thus transitioning the incorporation of nucleotides, which are detected by halogenated nucleotide specific antibodies. The transition from red to green marks the position of the replication fork at the time of switch from the first to the second nucleotide labels.

Growth kinetics was performed to determine the concentrations of the halogenated nucleotides used for labeling the PEL cells. Both, BCBL-1 and JSC-1 cells were cultured with 10, 25 and 50 µM of IdU and CldU for 12 h and the cell viability was determined. These halogenated nucleotides did not show any significant change in the growth pattern up to 25 µM (Supplemental Figure S1A and B). Therefore, we used 25 µM, final concentrations, of these analogs for labeling the cells in our subsequent experiments. The labeled cells were also analyzed for their cell cycle profiles, to determine that the cells were growing normally, by flow cytometric analysis after staining with propidium iodide (PI). These cells did not show any difference in cell cycle profile as compared to the untreated cells suggesting that these analogs did not affect the cell cycle at the concentrations used for labeling the cells (Supplemental Figure S1, C and D). We also determined whether an addition of nucleotide analogs triggers lytic reactivation by analyzing the levels of the immediate early gene, RTA (replication and transcriptional activator). RTA, which is essential for initiating the lytic DNA replication, was undetected by the western blot in IdU treated BCBL-1 and JSC-1 cells (Supplemental Figure S1E).

Labeled cells were washed to remove any unincorporated nucleotides and were resuspended then placed in agarose plugs. These cells were lysed as mentioned in the materials and method section followed by digestion to linearize the KSHV genome. The cells are digested in agarose plugs to avoid any DNA breakage, as we required full-length episomes for analysis. The linearized KSHV genome was partially separated from genomic fragments by pulsed field gel electrophoresis using the CHEF DRII system as described in methods section. The band of linear KSHV genomes in the gel was determined by a Southern blot using specific probes shown in the supplemental information (Supplemental Figure S2). Linear KSHV genome showed a band at approx 165 kb, which was determined based on pulsed field DNA markers (NEB). Agarose from the gel was excised for DNA extraction. The DNA was extracted after melting the agarose slice-containing the regions of interest and digesting with gelase. An aliquot of DNA solution was stretched between the positively charged (3-aminopropyl-tri-ethoxysilane-coate) glass slides and non-silanized cover slip through capillary action. Staining the DNA with an intercalating dye, YOYO-1, aided in visualizing stretched DNA by fluorescence microscopy (Supplemental Figure S3). Since molecules stretched by capillary action may vary in their orientation and size, three biotinylated probes were used for fluorescence in situ hybridization (FISH) followed by detection with Alexaflour 350-conjugated Avidin (shown in blue throughout the manuscript) to distinguish KSHV molecules from other DNA molecules. Use of different sizes probes produced a distinctive blue “bar code” which helped in determining the orientation of the molecules. The halogenated nucleotides are detected using specific monoclonal antibodies and secondary antibodies conjugated with Alexaflour 488 (shown in green throughout the manuscript; CldU) and Alexaflour 594 (shown in red throughout the manuscript; IdU).

**Figure 1:**
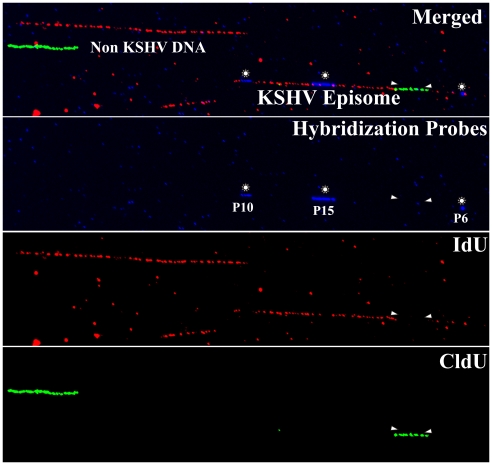
Immuno-staining and fluorescent hybridization of individual KSHV episomes. Image of three stretched DNA molecules in the same optical field. The hybridization signals (FISH probe, p10, p15 and p6) and the immunostaining to detect the halogenated nucleotides are shown in different pseudocolors (red  =  IdU; iodo deoxyuridine, green  =  CldU; chloro deoxyuridine and blue  =  hybridization probes). The top panel shows the merged image. The different color channels are shown separately in the lower panels. One of the above stretched molecules is a *Pme*I linearized KSHV episome which can be recognized by the presence of the hybridization signals. White arrowheads indicate the approximate position of the replication forks at the time of the switch from the first to the second labeling period.

**Figure 2:**
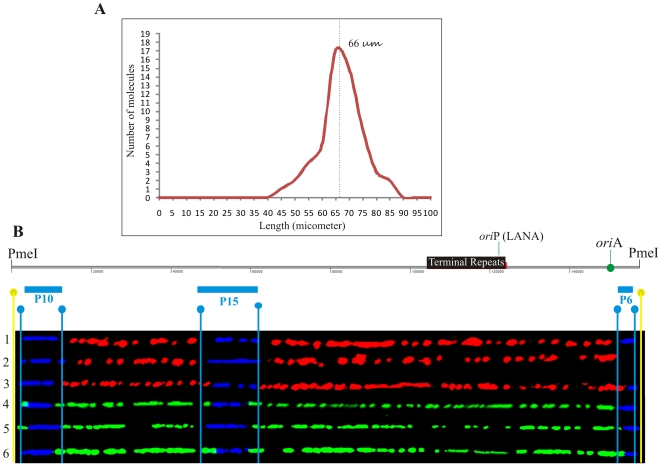
Stretching of DNA molecules on silanized glass slides by capillary force. A. KSHV episomes with recognizable hybridization signals were analyzed for their size. The majority (95%) of the molecules were between 66 µm (±10 µm) after stretching, which corresponds to about 2.5 kb/µm based on the 165 kb KSHV genome. Schematic of the *Pme*I-linearized BCBL-1 KSHV genome with the position of the terminal repeat (TRs). TR binds to Latency Associated Nuclear Antigen (LANA) and initiates replication in LANA dependent manner. Additional *ori* site (*ori*A) does not require LANA in trans to support replication. B. Images of six *Pme*I-linearized KSHV episomes aligned with the KSHV map after hybridization and immunostaining of the DNA molecules and digital adjustment of length. The FISH hybridization signals are shown in blue. Immunostaining to detect the halogenated nucleotides is shown by red and green. Vertical light blue lines indicate the positions of the ends of the hybridization probes and yellow lines, the position of the *Pme*I site used to linearize the KSHV episomes. All the molecules shown in this Figure represent KSHV episomes duplicated during either the first labeling period (Red) or the second labeling period (green).

Since the DNA molecules were substituted throughout their length with nucleotide analogs, they were easily detected even in the presence of substantial background signals. Figure 1 shows an optical field with halogenated nucleotide substituted DNA (linear red and green signals). The signals, which are not on a line, may have been due to the background signals or broken pieces of DNA. The KSHV DNA was distinguished from the non-KSHV DNA by the presence of FISH (blue) signals. In order to determine the average length of linear KSHV episomes, fully substituted molecules either with the first label, IdU (detected in red) or the second label, CldU (detected in green) were aligned with the schematic of the *Pme*I linearized genome. These molecules marked by the presence of probe signals, P10 at the left side and P6 at the right side (depicted in the schematic Figure 2B) of the *Pme*I linearized genome, showed an average length of 66 µm corresponding to about 2.5 kb/µm (Figure 2A). The molecules that substituted fully during first or the second labeling periods allowed us to calculate fork rates.

### BCBL-1 KSHV genome region replicates uniformly throughout the genome

**Figure 3:**
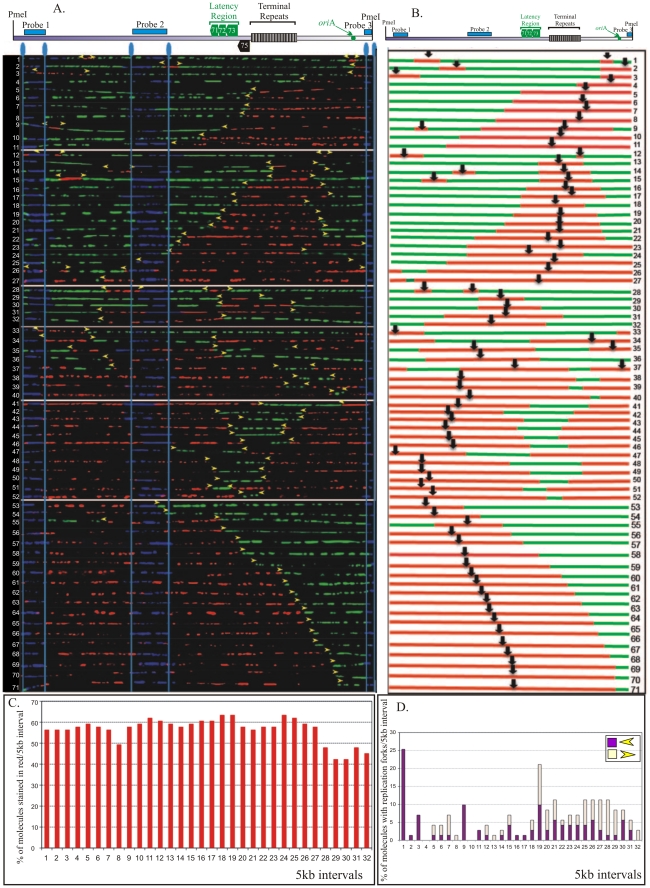
SMARD performed on *Pme*I linearized KSHV genome from BCBL-1 cells. A. *Pme*I linearized KSHV episomes were hybridized to detect the probe signal for alignment with genomic map. Molecules incorporating both halogenated nucleotides were assembled from 1-71 based on increasing staining of the first label shown in red. Vertical blue lines mark the position of hybridization signals. Yellow arrowheads indicate the approximate position of red-to-green transition. B. Schematic of molecules with red and green staining signals shown in panel A. The middle of the red stained signal represents the replication initiation site of a bidirectional replication. C. The replication profile of BCBL-1 KSHV episomes. Replication profile was obtained by vertically dividing the episome images at every 5 kb interval and determining the percentage of molecule stained with red in each interval. D. Profile of replication fork abundance and the direction of their movement throughout the KSHV genome. Presence of red-to-green transition sites was determining in each 5 kb vertical intervals for calculating the fork abundance. The vertical axis represents the percentage of molecules with replication fork in each 5 kb interval. The forks moving right to left are depicted in purple and the left to right moving forks are shown in light yellow.

In order to identify the replication initiation sites on the BCBL-1 genome, photomicrographic images of the molecules that were fully substituted and contained both nucleotide analogs were aligned using the FISH signals as a template. The alignment was done in a non-subjective manner but based on increasing incorporation of IdU (red signals) in randomly selected representative stretched DNA molecules from the pool of collected molecules. Alignment of molecules based on the increasing signals effectively removes any potential bias in the arrangement of molecules. Therefore, if a replication fork moves in a single direction within the examined region, the molecules will show an increase in the length of red signals from one end. A replication initiation site is characterized by a region of red signal (IdU sites) flanked by the green signals (CldU sites) on both sides. Additionally, if the replication fork moves in both directions, the molecules will have increasing red signal on both sides of the replication initiation sites. In contrast to replication initiation, the termination sites are marked by a patch of green stain flanked by the red signals on both sides. A schematic of replication initiation sites and termination are shown in Figure 3. Replication initiation sites are marked by black vertical black arrows (Figure 3B).

We collected images of 151 *Pme*I linearized KSHV episomes which incorporated the halogenated nucleotides along their entire length (41 fully substituted with IdU, 39 fully substituted with CldU and 71 stained with both IdU and CldU). The results of these experiments are shown in Figure 3 and supplemental Figure S4. The transition site from red to green in the molecules with both the halogenated nucleotides defines the position of replication fork at the time of transition from the first to the second label. The red staining portion of these molecules that varied in different *Pme*I linearized molecules suggested that replication initiated at different positions in these molecules. Schematics of the molecules with red and green staining patterns were drawn and center (vertical black arrow in diagrams and horizontal yellow arrows show direction of the replication forks) of the red stained patches were marked as the site of replication initiation assuming (as is usually the case) latent replication proceeds in a bi-directional manner (Figure 3A and B). In order to define the progression of replication throughout the genome, the replication profiles of these molecules were generated (Figure 3C). The replication profile was generated by dividing the KSHV genome map into 5 kb intervals and determining the percentage of molecules stained with red in each interval. The replication profiles are important in determining the region, which replicated first, termed as RRF (region replicating first) and regions, which replicated last, RRL. RRF are proposed to contain the replication initiation sites used most frequently for the duplication of the genome. However, in BCBL-1 episomes, approximately 60% of the molecules showed replication initiation within intervals 1 through 27. Intervals 28–31 showed slightly lower frequency of replication initiation (Figure 3C). Interestingly, the terminal repeats region (spanning intervals 22–25), containing multiple LANA dependent replication origins, did not show any preferential sites for replication initiation. This suggested that the KSHV genome could initiate replication throughout the genome. We also detected molecules with replication forks moving in only one direction (Figure 3A and B, molecules 56–71), which could be due to unidirectional replication in some molecules.

### Replication forks move without major pausing sites throughout the BCBL-1 genome

The progression of replication forks throughout the KSHV genome was described by the profiles of replication fork abundance, which was obtained by dividing the KSHV genome map into 5 kb intervals and then indicating the percentage of molecules with red-to-green transitions within each interval. As mentioned above, the transitions sites indicate the position and the direction of replication forks at the time of switching of the first to the second label [38]. Accumulation of transition sites from red to green at intervals 1, 3 and 9 indicated that the replication fork was not moving freely within those intervals which could suggest that the observed region may have a site where forks pause but only for a short duration.

Profiles of replication fork abundance were also used to determine the prevalent direction of replication fork movement throughout the KSHV genome (Figure 3D). In the BCBL-1 genome, the replication forks moved in both directions at similar frequencies in most of the intervals indicating bi-directional replication. However, intervals 27, 28, 29 and 32 showed higher number of molecules with forks moving towards the right end of the genome suggesting that the replication fork may have paused for a short time in that region (Figure 3D). Peaks with molecules having the replication fork movement in only one direction may indicate the replication fork pausing sites (Figure 3D). It could also be possible that the left end of the fork is masked with the probe (p10) or deleted during DNA processing in those molecules with single fork. Single replication fork could also be due to lytic replication, which utilizes a rolling circle strategy for replication. KSHV infected cells primarily maintains latent viral genome but a small proportion of the cells may undergo lytic reactivation spontaneously thus leading to the generation of the molecules with a single fork. Nonetheless these molecules did not appear to significantly affect the calculation of the replication fork progression.

### Replication initiation was not limited to the TR region

Earlier studies mapped the replication initiation site to the terminal repeat region and to the autonomously replicating element region (*ori*A) [28], [36]. However, these studies were performed only on selected regions of the genome without taking the complete genome into consideration. SMARD detects replication initiation sites throughout the genome by analyzing the immunostaining patterns of the DNA molecules. Multiple initiation events should produce multiple red staining patches each surrounded by green signals. The immunostaining pattern of these molecules (shown in Figure 3B) revealed multiple replication initiation sites. For example, early initiation events took place within the TR region of molecules 9, 14 and 15 detected by red stained region flanked by the green signals (Figure 3B). However, shorter red regions were also detected on the same molecules indicating the occurrence of replication initiation events at later time points. We indicated that multiple replication initiations were present if replication initiation events on particular molecules were not synchronous (different sizes of red staining patches), we referred the primary to the first initiation event and secondary to the subsequent initiation events. The secondary initiation event in the TR region is shown by a smaller red staining signal in molecule 12 (Figure 3B). In molecules 1, 2, 35 and 37 multiple initiation events were recorded with secondary initiation events in region of *ori*A (Figure 3B). Secondary initiation events were also detected within regions of genes transcribed during latent infection. Molecule 28 has a secondary initiation event in the *v*IRF transcripts region. This suggests that initiation events were not limited to any particular region of the genome but that the entire KSHV genome constitutes a large initiation zone for replication.

### DNA replication proceeds at different speeds throughout the KSHV genome

**Figure 4:**
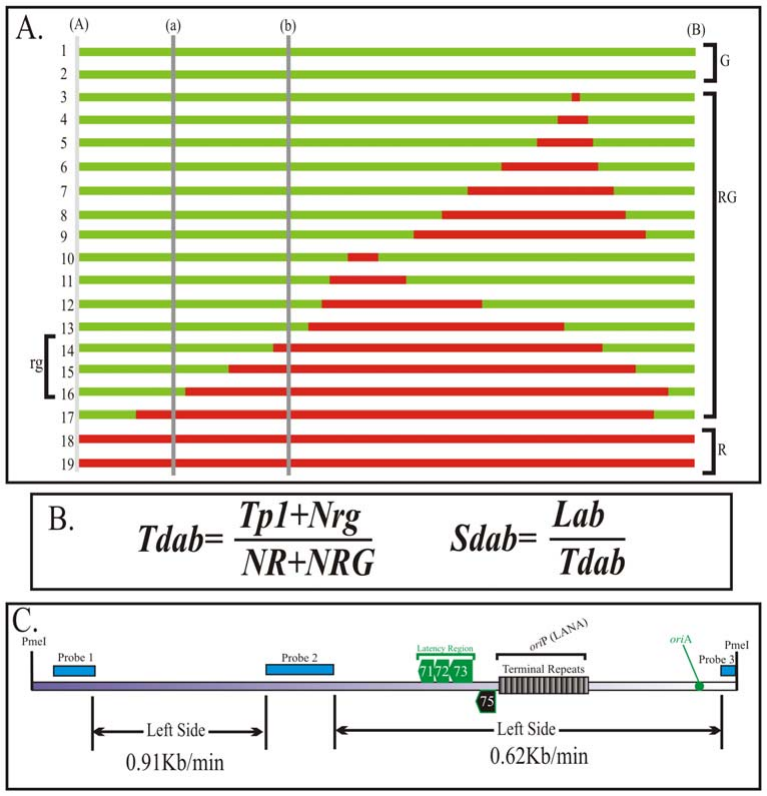
Calculation of the duplication speed of KSHV genome regions. We used previously published procedure to calculate the duplication speed (Td) of different genomic regions. A. Schematic shows how to calculate duplication speed of any specific region with an example of Td calculation between regions (a)-(b) using information obtained from entire length replication profile. Nineteen hypothetical fully labeled molecules, with either single or both halogenated nucleotides, are shown in red and green colors. The molecules, which started and duplicated during first labeling period are depicted as fully red (R) and the one that completed their replication during second labeling period are shown in green (G). The molecules, which started their replication during first labeling period but completed during second labeling period are shown to have both red and green (RG). The numbers of red and green molecules, in the pool of total molecules, are proportional to the time required to replicate the region between (A)- (B). Similarly, the number of rg molecules depends on the time required to replicate the region (a)-(b). B. Equation to calculate the duplication time required for replicating the region (a)-(b). Tp1 is time used for first pulsing, which was 4 h for these experiments; N_R,_ Number of Red (R) molecules; N_RG_, Number of RG molecules; N_rg_; Number of rg molecules. The speed of fork movement, Sd_ab_ is calculated by dividing the distance (size) between the specified regions with the time (Td_ab_) required for the duplication of the specified region (a) and (b). C. Duplication speed of BCBL-1 KSHV genome, calculated based on the molecules shown in Figure 3 and supplemental Figure S4. The duplication speeds of region marked by double-headed arrows are indicated.

In previous experiments we showed replication initiation sites and the progression of replication fork qualitatively. Here we wanted to determine the speed of replication forks in different regions of the KSHV genome using the equation described earlier [38]. SMARD data is used to determine the average time required for duplication of any portion of the genome (*T*d: Figure 4A). By using the time required for the duplication of a known length or segment, duplication speed (*S*d) can also be calculated (Figure 4B). These conclusions are based on the data from a large pool of molecules and are performed on large genomic segments. Therefore the quantitation is not significantly affected by the resolution of the technique. We calculated the duplication speed of the left and the right side of the KSHV genome using the fully substituted red and green molecules (Supplemental Figure S4). We found that these regions replicated at a different speed with the left side proceeding at 0.91 kb/min and 0.62 kb/min in the right side of the molecules (Figure 4C). The differences in the speed could be due to the complexity (high GC content due to multiple TR units) on the right side of the genome, which may impede the movement of replication forks.

### Multiple initiation events can take place on a single KSHV genome molecule

As mentioned previously, some molecules in the aligned image showed multiple red stained patches flanked by green patches indicating the occurrence of multiple initiation events (Figure 3B). Two replication initiation sites were detected in 10 out of 71 imaged molecules suggesting that multiple replication events significantly occurred significantly during replication. These initiation events are distinctly separated in terms of distance and time of their activation. However, replication initiation events that are located near each other may have not been detected as individual initiation sites due to the resolution limit (approx. 5000 bp) of this technique. The terminal repeat region, which consists of approx. 25 copies of terminal repeat units may be initiating replication at each unit since the individual units are shown to have functional replication elements [24]. It would be interesting to perform SMARD on the TR region cloned into a replication deficient vector (cosmid) to determine whether replication initiated at all the TR units or is restricted to any specific one. Based on the SMARD data performed on the entire genome we confirmed the occurrence of multiple replication events on the BCBL-1 genome.

### BCBL-1 KSHV accumulates components of the replication machinery at various sites on the genome

Initiation of DNA replication requires assembly of pre-replication complexes (*pre*-RCs) on DNA [41], [42]. Assembly of the *pre*-RC begins with the binding of origin recognition complexes (ORCs) to the chromatin [41], [42], [43]. ORCs consist of a stable core complex including subunits ORC2-ORC5 associated with less stably bound components ORC1 and ORC6 [42], [44], [45]. These ORCs recruit two other proteins, Cdc6 and Cdt1, which stabilizes the binding of ORC and allows Cdt1 to load the replicative helicase, mini-chromosome maintenance (MCMs) proteins (MCM2-7) [46], [47], [48]. Association of ORCs, Cdc6, Cdt1 and MCM2-7 completes the process of pre-RC assembly, also referred to as licensing of the replication origin. Importantly, pre-RC formation and replication competency is restricted to the G1 phase of the cell cycle [46], [49]. Some replication origins are activated at the beginning of S-phase by the recruitment of protein kinases, Cdc7 and Cdk2 [46], [49]. Therefore, chromatin immunoprecipitated from G1/S cells with Pre-RC complex proteins brings down the active replication initiation sites. These pre-RC components are disassembled after the cell completes the cell division. We used ORC2 and MCM3 (components of *pre*-RC) antibodies to immunoprecipitate chromatin from G1/S cells and compared their association with G2/M phase cells. In order to get pure population of cells in G1/S and G2/M phases, we used the centrifugal elutriation on asynchronously growing cells. The cell cycle analysis of these cells by propidium iodide showed that over 80% cells were in G1/S phase (Supplemental Figure S5). Similarly, the G2/M phase cells had very high proportion of cells in the fraction used for ChIP assay (Supplemental Figure S5).

**Figure 5:**
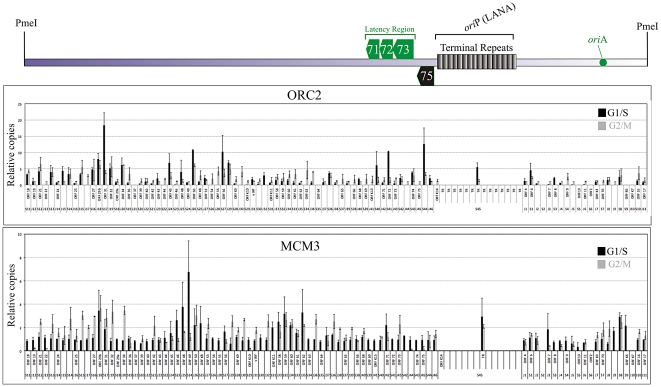
Chromatin Immunoprecipitation from G1/S and G2M enriched BCBL-1 cells using ORC2 and MCM3 antibodies. Actively growing BCBL-1 cells were subjected to centrifugal elutriation to get pure populations of G1/S and G2/M phase cells. ChIP was performed as described in the methods using anti-ORC2 and anti-MCM-3 antibodies. Purified DNA was used as template for the amplification of KSHV genome at every 1–2 kb. Relative amplification was calculated by subtracting the amplification with the control antibodies.

Immunoprecipitated chromatin from the G1/S and G2/M phase cells was analyzed for relative amplification of immunoprecipitated DNA in a semi-quantitative real-time PCR assay. We analyzed the entire BCBL-1 genome by using PCR primers at approximately 1.5 kb intervals. Comparison of immunoprecipitating copies of these genomic regions in G1/S and G2/M phase cells showed very specific association of both ORC2 and MCM3 in G1/S cells (Figure 5). Importantly, there were multiple sites on the genome, which were enriched with ORC2 and MCM3 chromatin immunoprecipitation suggesting the formation of pre-RC at those sites (Figure 5). This supports our SMARD data of multiple replication initiation sites on the genome. Regions around ORF6, ORF31, ORF50, ORF56, ORF71-71 and ORF75 showed relatively higher copies of genome precipitating with ORC2 in G1/S cells. The enrichment of these fragments was also seen with MCM3 antibodies suggesting the accumulation of *pre*-RCs at multiple sites on the KSHV genome. Chromatin immunoprecipitating the TR region (multiple reiterated copies of terminal repeats), represented by a primer set S45, showed specific enrichment of ORC2 and MCM3 in G1/S phase cells suggesting cell cycle specific accumulation of *pre*-RCs and origin firing at the TR.

### JSC-1 cells showed slightly different replication initiation pattern

**Figure 6:**
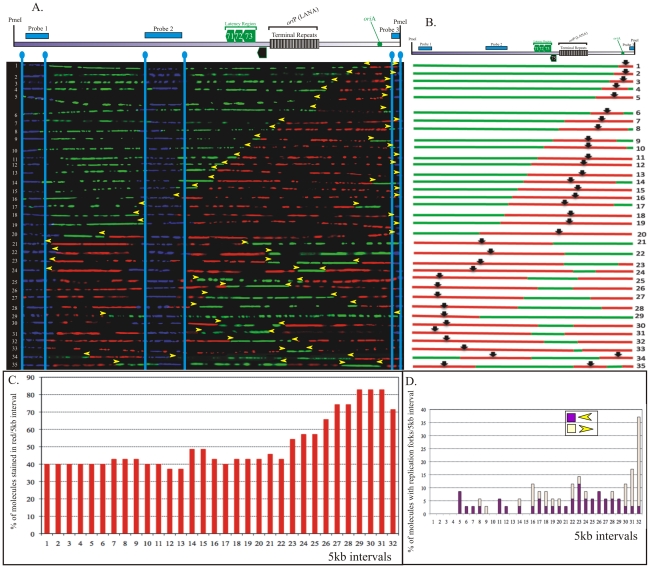
SMARD on JSC-1 KSHV episomes. A. Images of 35 *Pme*I linearized JSC-1 KSHV molecules, labeled with both halogenated nucleotides are shown with the schematic of KSHV genome. The molecules were organized based on the increasing red stained region on individual molecules. B. Black arrows indicate the position of replication initiation sites represented on the schematic of the molecules. C. Replication profile of the *Pme*I linearized episomes shown in panel A. As described earlier, replication profile was obtained by dividing the genome at every 5 kb interval and calculating the percentage of red molecules in each interval. D. Profile of replication fork abundance and the direction of replication fork movement. Graphical representation shows the percentage of replication forks in each 5 kb interval. The forks moving right to left are depicted in purple and the left to right moving forks are shown in light yellow.

We were interested in analyzing the KSHV genome replication in another PEL cell line. We selected JSC-1 cells because this cell line maintains higher copies of the viral genome in latently infected cells. In order to determine whether the difference in copy number was due to the number of replication initiation events or the speed of the replication fork movement? We performed SMARD similar to that done for the BCBL-1 genome and recovered the images of 72 *Pme*I linearized KSHV episomes substituted along their entire length with halogenated nucleotides (35 red/green, 19 red and 18 green). The molecules with red and green were aligned with increasing red staining signals as shown in Figure 6. These two KSHV strains, BCBL-1 and JSC-1 showed a slight difference in the replication initiation patterns. In BCBL-1 KSHV genome, replication initiation events occurred uniformly throughout the entire length but in JSC-1 KSHV the right end of the genome showed higher replication initiation events (Figure 6C). JSC-1 KSHV also showed multiple initiation events but was slightly lower compared to BCBL-1 (2 molecules in JSC-1 compared to 10 molecules in BCBL-1 images). These multiple initiation events were mostly asynchronous as determined by different sizes of the regions with red staining (Figure 6 A and B). The replication profile of JSC-1 KSHV also showed the difference in termination sites. However, the genome replicated uniformly without significant pausing (Figure 6D). In summary, these data showed that replication initiation sites are not confined to any specific region of the KSHV genome and thus led us to conclude that the utilization of replication initiation sites can change in different molecules as well as different strains of KSHV. This result also suggests that replication initiation may also be controlled by epigenetic modification and will be an important area of study.

### The duplication speed of the left portions of the KSHV genome varies in BCBL-1 and JSC-1 cells

**Figure 7:**
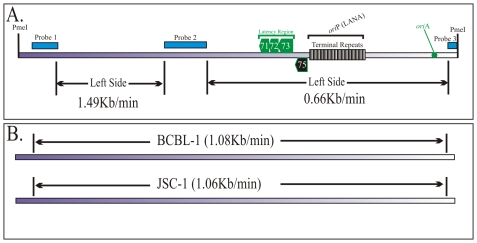
Duplication speed of JSC-1 KSHV genome. A. Duplication speed of JSC-1 genome was calculated using molecules from Figure 6 and supplementary Figure S6. B. Duplication speeds of KSHV genomes of BCBL-1 and JSC-1 cell lines. The duplication speed of entire genome was calculated based on the images substituted with both halogenated nucleotide of Figure 3 and Figure 6 and singly substituted images of supplemental Figures S4 and S6. The time used for pulsing with the first and second label was 4 h during each labeling period.

Since the replication initiation sites of KSHV in BCBL-1 and JSC-1 cells were slightly different, we wanted to determine if the speed of DNA replication was also different. Images of the molecules shown in Figure 3 and the supplemental Figure S6 were used for calculating the *S*d for the left and right portion of the JSC-1 genome. We found that DNA duplication proceeded at different speed in the left verses right portion of the genome with 1.49 kb/min being highest in the left side and 0.66 kb/min in the right side (Figure 7A). It was interesting to note that the right portion of the genome, which has terminal repeat region, replicated with almost similar speed, 0.66 and 0.62 kb/min in both BCBL-1 and JSC-1 KSHV genome, respectively. However, the left end of the genome duplicated much faster in JSC-1 KSHV (1.49 kb/min) as compared to the BCBL-1 KSHV (0.91 kb/min). This suggests that replication progresses with different kinetics within different regions of the genome and could be due to genomic complexity or due to the presence of different pausing sites. Additionally, duplication may also be affected by the presence of multiple initiation events within the region replicating faster.

### KSHV genomes of BCBL-1 and JSC-1 cells replicated at similar rates

The duplication speed, which reflects the average number of replication forks for the replication of specific region, was determined for the entire KSHV genome. Although the duplication speeds of the left portion of the BCBL-1 and JSC-1 virus were different the overall speed for the duplication of the entire KSHV genome was similar. Duplication speed of BCBL-1 KSHV was 1.08 kb/min and JSC-1 duplicated with a speed of 1.06 kb/min (Figure 7B). On average the duplication speed of KSHV in both PEL cells were 1.0 kb/min, which was slightly slower compared to the duplication speed of Epstein Bar Virus (EBV) (1.5 kb/min) [38].

## Discussion

The KSHV genome persists in the infected cells in a latent state by expressing a small number of viral genes [1], [2]. Furthermore, LANA is a predominantly expressed protein in all KSHV infected cells [2], [50], [51]. LANA has been shown to tether the viral genome to the host chromosome by binding to the histone proteins [5], [21]. Further, LANA has been shown to be critical in maintaining the viral genome copies in these cells as a recombinant KSHV (BAC36) deleted for the LANA gene could not establish latent infection and was lost from the cells within two weeks [20]. Additionally, LANA depletion by shRNA reduced the viral genome copies to basal level in PEL cells [52]. These studies suggested a role for LANA in maintaining the viral genome in infected cells. Since LANA colocalized with the KSHV genome on the host chromosome, it was suggested that LANA binds to the viral genome directly [5]. Studies focusing on the identification of LANA binding sites identified the LANA binding sites along with the replication elements within the terminal repeat region [22], [28]. Further studies, focused on the identification of replication initiation sites, identified the LANA dependent origin within the terminal repeat region [23], [24], [26], [53]. In our more recent study, we analyzed the left end of the KSHV genome and identified a replication origin (*ori*A), which did not require LANA expression in *trans* [36]. This prompted us to analyze the entire KSHV genome for replication initiation events using a recently developed and powerful technique, SMARD, that looks at the replication events on individual DNA molecules [38].

In this study, we determined how DNA replication initiated and progressed on KSHV episomes of two latently infected PEL cell lines (BCBL-1 and JSC-1). We further determined the abundance and the dynamics of replication fork movement in these cells. Replication can initiate at various regions of the genome including the terminal repeat region. There were slight differences in the direction of replication fork movement and the duplication speed of the left end of KSHV genome in BCBL-1 and JSC-1 cells (Figure 3 and 6). However, the frequency of replication initiation and termination events varied across the genome and between the two KSHV strains. These differences were detected even though the genomic sequences of these two strains are identical. SMARD performed on two different strains of EBV also showed a difference in the replication initiation and termination sites [38]. Differences in replication profiles were proposed to be due to the differences in genetic makeup of these two strains (MutuI has deletion of internal repeat 1 and Raji has two short deletions) [38]. However, analysis of various regions by SMARD and 2D gel analysis confirmed that these deletions did not account for all the differences in the replication pattern [38].

This study also characterized the pausing sites of the replication forks outside the TR region. Accumulation of replication forks is clearly detected in both the strains of KSHV within the genomic regions outside the TR and at the left end of the genome. Detection of lower numbers of pausing sites within the left end of JSC-1 could contribute to a faster replication fork movement within that region. Speed of replication within the right side of the genome did not vary significantly suggesting the presence of similar epigenetic and genetic organization in that region within these two KSHV strains. Since transcription may have an essential role in controlling replication fork movement and pausing, it may suggest that both strains have similar transcriptional patterns within the right end of the genome. Another common feature between BCBL-1 and JSC1 was that the time required for the replication of the genome was similar. This suggests that replication forks proceeded uniformly throughout the genome to complete the replication.

The presence of single replication forks in some molecules may indicate a replication pausing zone at one end of the bi-directional replication fork or the fork may have got masked in the FISH probe signal at the left end of the genome. Detection of single replication fork may also indicate that some molecules were undergoing unidirectional replication as proposed for the rolling circle mechanism. Herpesviruses replicate by a rolling circle mechanism during lytic cycle using lytic origins of replication [54]. Therefore, it could be possible that a small proportion of the cells BCBL-1 and JSC-1 may have undergone lytic reactivation and produced these single replication fork-containing molecules. It would be interesting to determine the lytic replication mechanism of KSHV using SMARD but that is beyond the scope of this study.

Chromatin immunoprecipitation data with ORC2 and MCM3 identified multiple sites where the *pre*-RCs were accumulated suggesting the formation of multiple active replication initiation sites. However, it is important to note that *pre*-RCs sites on the genome and replication initiation by SMARD showed very limited correlation. This could possibly be due to the fact that the SMARD data is from individual genome molecules as compared to ChIP data, which is a cumulative data from million copies of the KSHV genome. The genomic region between primer sets S16-S16 (Figure 5) showed enrichment of *pre*-RCs, which also correlated with replication initiation sites determined by SMARD (interval 5 of Figure 3D). Importantly, epigenetic modifications may also have a critical role in determining the replication initiation sites. Studies have shown that histone acetylation can influence the timing of replication origin firings [55]. A recent study showed comprehensive epigenetic modifications of histone H3 for acetylation, K4 and K9 tri-methylation and K27 tri-methylation [56]. Histone H3 acetylation and tri-methylation at K4 indicates a transcriptionally active chromatin. Chromatin immunoprecipitation with acetylated histone H3 and tri-methylated Lysine 4 (K4) in KSHV PEL cells, BCBL-1 has shown various regions with active chromatin [56]. Also studies have demonstrated that regions with active transcription shows reduced or no replication as both of these activities do not occur at the same time in mammalian cells [57]. Presence of multiple silent chromatin regions in the BCBL-1 genome could possibly explain the occurrence of multiple replication initiation events, and the pausing of replication forks on the latently replicating genomes. Each of the transcriptionally silenced regions may potentially serve as the replication initiation sites and thus were detected by the SMARD assay. Additionally, the replication fork stalling sites could be the regions where replication forks traversed through the RNA polymerase bound DNA site as the RNA polymerase may remove or inactivate the pre-replication complex deposited on the DNA for replication. However, understanding the coupled events of replication and transcription may be very complex mechanistically and would be beyond the scope this study. A recent study on bacterial genome replication understanding the movement of replisome through a protein nucleic acid block showed that head on collision of the replisome with RNA polymerase results in replication fork arrest but co-directional collision has little or no effect on the fork progression [58]. Therefore, the presence of transcriptionally active sites on the genome may be the reason for replication fork pausing if the replisome collides head on with the transcription machinery.

In terms of the replication elements, the terminal repeat elements are well characterized for the replication of plasmids in the presence of LANA [23], [28]. SMARD also detected replication initiation sites in the TR region but showed that this was not the preferred site for genome duplication. The infrequent usage of TR as the replication site does not seem to be due to the lack of *pre*-RCs accumulation in this region. Various studies have shown the recruitment of ORCs and MCMs in the TR region suggesting functional replication by the TR region [33], [34]. However, the role of TR mediated replication in the context of entire genome was not determined. Therefore, it would be interesting to generate a recombinant KSHV lacking the replication origin site within the TR element to evaluate its potential for replication and establishment of latent infection. There are technical difficulties to generate a recombinant KSHV deleted for RE element (element required for replication initiation in the TR region) because each TR copy has one RE element and there are 25–30 reiterated TR copies [24]. Therefore, it will require multiple rounds of recombination to remove the RE elements from each TR copy. Alternatively, a recombinant KSHV can be generated by excising all the TR copies and replacing it with RE deleted TRs in the BAC KSHV (BAC36) that will lack the TR mediated replication. Hence, this recombinant KSHV will be important in evaluating the role of primary and secondary replication initiation sites in the establishment of latent infection.

This study suggests that the KSHV genome replicates not only by using the cellular replication machinery but also follows a mechanism similar to that used for the cellular DNA replication. DNA replication in mammalian cells initiates in replication zones, which are similar to the replication zone of the entire KSHV genome [59]. This shows a broad impact of our study since KSHV can be used as a model system to investigate the unresolved molecular mechanism of cellular DNA replication. This study also highlights the divergent evolutionary trends of human viruses where large dsDNA viruses replicate using a similar mechanism to the host cellular DNA without requiring viral proteins which could be detected by the host immune system. Use of a similar replication mechanism by these viruses may provide an advantage in establishing life long infections without being detected by the host immune surveillance even in healthy individuals.

## Methods

### Cell cultures, KSHV strains and double labeling of replicating DNA

KSHV infected body cavity based lymphoma cells (BCBL-1 and JSC-1) were cultured in RPMI supplemented with 10% fetal bovine serum, 2 mM L-glutamine and penicillin-streptomycin (5 U/ml and 5 µg/ml, respectively). These cells were grown at low density (3×10^5^–6×10^5^) in order to keep the virus primarily in the latent state. Cells growing at approximately 5×10^5^ cells per ml were labeled for 4 hrs each with 25 µM 5′-iodo-2′-deoxyuridine (IdU; first label) and 25 µM 5′-chloro-2′-deoxyuridine (CldU; second label). IdU was directly added into the media with actively growing cells followed by low speed centrifugation (600 xg) at RT to collect the cells at the end of first labeling period. These cells were washed with warm PBS and resuspended in pre-warmed media with a second label, CldU at the density of 5×10^5^ cells/ml. Cells were collected, washed and mixed with molten InCert agarose (Lonza Inc. Rockland, ME) and poured into the molds for making plugs. These plugs contained approximately 10^6^ cells/plug.

### Extraction of linear KSHV genome from the agarose plugs

Plugs containing BCBL-1 and JSC-1 cells were treated with cell lysis buffer (0.5 M EDTA+1% Sarcosine and Proteinase K) at 50°C for 96 h by changing the lysis buffer at every 24 h. These plugs were thoroughly washed with TE and digested to linearize the KSHV genome; we chose *Pme*I restriction enzyme, which cleaves the viral genome once. Before digestion with *Pme*I, the plugs were incubated with digestion buffer and digested with 50 U of enzyme.

Digested plugs were loaded onto 0.6% LMP agarose (Seplaque, Lonza Inc., Rockland, ME) followed by resolving them on a pulse field gel electrophoresis using CHEF-DRII (Bio-Rad Laboratories, Hercules, CA). The region of linear KSHV genome banding on the gel was identified using KSHV specific ^32^P labeled probe after transferring part of the gel onto a nylon membrane. The linear KSHV genome was extracted from the gel by cutting a small slice of agarose containing KSHV DNA and treating with gelase (Epicenter Biotechnologies Inc., Madison, WI) as per manufacturer's recommendations.

### Stretching DNA molecules on glass slides

We used a capillary method to stretch DNA molecules on the glass slides, described previously [37], [38]. Isolated and enriched linear KSHV genome, after gelase treatment, was gently deposited at the interface between silanized microscope slides and non-silanized coverslips. The stretching was determined under a fluorescent microscope by adding DNA staining dye, YOYO-1 to the DNA mix before stretching. DNA was diluted to a point where there were no overlapping DNA molecules. The coverslips were removed gently followed by dipping the slides in methanol. DNA molecules were denatured with 0.1 M NaOH and fixed with Glutaraldehyde.

### Hybridization of KSHV genome with specific probes and immunostaining of the individual DNA molecules stretched on glass slides

Hybridization was performed as described earlier [38] using probes labeled with biotin-16-dUTP (Roche Inc. Indianapolis, IA). Three different sizes probes, p10 (10 kb region between 36883-47193), p15 (15 Kb region between 85820-100784) and p6 (6 kb region between 26937-33194 nt) corresponding to KSHV genome (accession number NC_93331) were used in this study. The above-mentioned regions of the KSHV genome were cloned into plasmid/cosmid vectors and sequenced to ensure the presence of desired region. A purified band excised from the vectors were labeled with biotin using bio-nick labeling kit (Invitrogen, Inc., Carlsbad, CA) according to the manufacturer's recommendations. The length of the probe varied from 100–500 bp as determined by agarose gel electrophoresis. The hybridization signals were detected by NeutrAvidin conjugated to Alexa Flour 350 (Molecular probes Inc.Eugene, OR) as described earlier [38]. Briefly, five layers of Alexa Flour 350 conjugated with NeutrAvidin and Biotinylated anti-Avidin antibodies (Vector Laboratories Inc., Burlingame, CA) were deposited on the microscopic slides after washing with PBS containing 0.03% Igepal CA-630 (Sigma Aldrich, St. Louis, MO) after each step. The purpose of the probe was to identify KSHV genome molecules from the pool of DNA molecules, including cellular DNA. By using asymmetric probes, we aligned the molecules in the same orientation, post-image capture. Since the cells were labeled for sufficient time (4 h each label) to replicate the entire KSHV genome, we could easily detect the KSHV molecules even in the presence of substantial hybridization background (blue dots in hybridization probes panel of Figure 1). The hybridization background does not affect the SMARD, therefore digitally removed in further Figures (Figure 2, 3, 6 and supplemental figures 4 and 6).

Incorporated IdU and CldU were detected by immunostaining of the stretched DNA along with the detection of biotinylated hybridization probes. The labeled IdU and CldU were stained with mouse anti-IdU and rat anti-CldU as primary antibodies, respectively. Alexa Flour 594 conjugated goat anti-mouse and Alexaflour 488 chicken anti-rat were used as secondary antibodies to detect IdU and CldU, respectively. As described previously [38], antibodies for IdU and CldU did not cross-react and also did not stain any unlabeled DNA. The slides were mounted and stored in the dark. The images were captured using an automated fluorescent microscope (Axio Observer, Zeiss Inc., Thornwood, NY) with 63X objective. The slides were scanned to find the signals for probe (Blue color) to ensure the intactness of the KSHV molecules. DNA molecules with either red, green or both colors with the hybridization signals were imaged. The lengths of the DNA molecules were measured after capturing the images using an inbuilt feature of image capture and analysis software (Axiovision, Zeiss In., Thornwood, NY). Molecules with all three probes signal and appropriate size were subjected to analysis. Individual molecules were cropped and aligned based on the content of increasing red signal from top to bottom (molecule number 1 through 72 in Figure 3 and 1 through 35 in Figure 6).

### Advantages of dual labeling procedure for the SMARD

As described previously [38], we used exponentially growing asynchronous cells to label the replicating DNA. Additionally, we used labeling periods required to fully replicate the KSHV genome to ensure that the replicating molecules were duplicated as the duplication time could vary in different molecules. We examined the molecules, which were fully substituted during these labeling periods and the molecules that have just started to replicate at the time the pulse incorporates throughout DNA molecule. However, the molecules, which had a replication fork in the middle of the molecule at the time of pulsing, will have two labels. Another set of cells, which starts replication at the time of second pulse, will have first label incorporated throughout the molecule and small patch of second label at the site of replication origin. The site of transition from the first label to the second marks the site of the replication fork. By analyzing these fully substituted molecules, we eliminated the bias of looking at replication forks at any particular region of the molecule. Therefore, our analyses faithfully represent the distribution of the replication fork in a steady state population of replicating molecules [38].

The use of long labeling periods to substitute the region of interests with halogenated nucleotides is advantageous because it provides an internal control, which cannot be achieved by short labeling periods. Since the molecules are fully immunostained, they are easy to align with the KSHV genomic map. This also allows us to detect the unevenly stretched molecules, which were discarded from further analysis. Since we look for contiguous staining in fully substituted molecules, any breakage detected by loss of signal can be easily detected. Another shortcoming, which we encountered during stretching of the DNA molecules, is overlapping signals of the DNA molecules. Since the molecules are fully substituted, these overlaps are easily detected so these are also discarded from the analysis. It is also important to mention that a hybridization signal reduces the affinities of IdU and CldU antibodies to the corresponding regions of the DNA molecules (Figure 1). This causes a significant loss of information in regions to which the probe hybridizes but ensures that the immunostained molecules we have analyzed are indeed the molecules of interest. Lastly, the dual labeling scheme and detection of both nucleotides allowed us to believe that these labeled cells replicated normally without the introduction of any bias in image collection. In an ideal condition, when the above-mentioned criteria are met, the number of molecules fully substituted with first label, IdU are expected to be similar to the number of CldU substituted molecules. These controls represent the steady state replication of KSHV DNA molecules.

### Chromatin immunoprecipitation assay

G1/S and G2/M fraction of the asynchronously growing BCBL-1 and JSC-1 cells were fractionated by centrifugal elutriation. Cells from these fractions were cross-linked with 1% formaldehyde by rocking for 10 min at RT followed by adding 125 mM glycine to stop the cross-linking reaction. Nuclei from these cells were isolated followed by sonication of the chromatin to an average length of 600 bp. Cell debris was removed by centrifugation of the chromatin at high speed for 15 min at 4°C. Samples were pre-cleared with salmon sperm DNA/ProteinA Sepharose slurry for 30 min at 4°C with rotation. Supernatant were collected after brief centrifugation. 10% of the total supernatant was saved for input control. The remainder, 90% was divided into three fractions. i) Control antibody [Sigma Aldrich St. Loius, MO]. ii) Anti-ORC2 [Santa Cruz Biotechnology, Inc., Santa Cruz, CA] and iii) anti-MCM3 antibody [Abcam Inc., Cambridge CA]. Immune complex was precipitated using salmon sperm DNA/ProteinA/ProteinG slurry. Beads were then washed consecutively and the complex was eluted by using the elution buffer [1% SDS/0.1 M NaHCO3] and reverse cross-linked by adding 0.3 M NaCl at 65°C for 4–5 h. The eluted DNA was purified by treatment with proteinase K at 45°C for 2 h, phenol extraction and ethanol precipitation. Chromatin saved for input was also reversed cross-linked to extract DNA for real-time PCR analysis. Purified DNA from input, control antibody, ORC2 and MCM3 were dissolved in 100 µl sterile water. Primers spanning the entire KSHV genome at every 1.5 kb were used in the real-time PCR assay. List of primers and their sequences are available upon request. DNA from input, control IgG, ORC2 and MCM3 samples were used from each primer set. Relative amounts of DNA in chromatin bound to ORC2 and MCM3 proteins were calculated by subtracting the amplification with control antibody.

### Centrifugal elutriation and cell cycle analysis

Centrifugal elutriation was performed essentially as previously described [60]. Briefly, BCBL1 and JSC-1 cells grown in RPMI 1640 at 5×10^5^ cells/ml were separated into different cell cycle phases, with the flow rates of 14, 16, 18, 20, 24, 29 and 36 ml/min by a modified Beckman JE 5.0 rotor (Beckman Coulter Inc., Brea, CA) The relative DNA content of the different fractions was determined by flow cytometry (Becton Dickinson Inc., Franklin Lakes, NJ) by the fluorescence intensity of propidium iodide stained cells. The fractions of G1 (16 ml/min) and G2 (29 ml/min) were performed for ChIP assay as described previously [61].

## Supporting Information

Figure S1 **Halogenated nucleotide analog did not affect the growth patterns of KSHV infected Cells.** A: Growth kinetics of BCBL-1 cells with various concentrations of IdU. B: Growth kinetics of JSC-1 cells with various IdU concentrations. C and D: Cell cycle profile of BCBL-1 and JSC-1 cells, respectively, with (10 and 25 uM) and without (Untreated) IdU pulsing. Pulsing with the nucleoside analog (IdU) did not significantly affect the growth of growth of the PEL cells (BCBL-1 and JSC-1). E. BCBL-1 and JSC-1 cells were treated with either 1 mM sodium butyrate (Histone Deacetylase inhibitor used for induction of lytic cycle) or 30 uM of IdU (halogenated nucleotide analog used for labeling) for 24 followed by detection of immediate early protein, RTA (replication and transcriptional activator) in a western blot assay. Levels of RTA were compared in un-induced (UN), Sodium butyrate (NaB) and in IdU treated (IdU) cells. IdU treatment did not show detectable levels of RTA. Anti-GAPDH antibody was used to show that the levels of total proteins were similar in all three samples.
(TIF)

Figure S2 **Pulse Field Gel Electrophoresis (PFGE) to isolate linear KSHV genomes.** Agarose plugs containing labeled cells (IdU and CldU) were digested with PmeI to linearize the genome. These plugs were loaded onto a low melt agarose gel along with the pulse field DNA marker (M on both side of the plugs) and resolved by a pulse field gel electrophoresis. Gel containing the central portion of plugs (grey part of EtBr gel) was excised and stored for KSHV genome extraction. Both the sides of the gel (labeled as EtBr gel) were Sothern transferred and probed to detect the KSHV genome. Linear KSHV genome signals detected (165 kb signals on Southern blot) were aligned with the central portion of the gel to excise the KSHV genome by gelase treatment.
(TIF)

Figure S3 **Visualization of stretched DNA on 3-aminopropyl-tri-ethoxysilane coated slides.** BCBL-1 DNA extracted by the pulse field gel electrophoresis was stained with YOYO-1 dye to visualize after stretching the DNA by capillary force. The stretched DNA was imaged at 60X oil objective using Zeiss fluorescent microscope.
(TIF)

Figure S4 **Fully substituted BCBL-1 KSHV genome.** KSHV genome molecules replicated in BCBL-1 cells labeled with either the first label (A, Red) or the second label (B, Green). We collected forty one fully red and thirty nine fully green molecules while imaging molecules labeled with both the analogs. These molecules were used in the calculation of replication fork meovement.
(TIF)

Figure S5 **Cell cycle profiles of the elutriated BCBL-1 cells used in ChIP assay.** Fraction 16 (BCBL1-16) which constituted over 80% cells in G1/S phase and fraction 29 (BCBL1-29) with over 80% cells in G2/M phase were used in ChIP assay.
(TIF)

Figure S6 **Fully substituted JSC-1 KSHV genome.** KSHV genome molecules replicated in JSC-1 cells labeled with either the first label (A, Red) or the second label (B, Green). There were images of nineteen fully red and eighteen fully green molecules in JSC-1 cells.
(TIF)
