# Supplementary figures and images for: Single Molecule Analysis of Replicated DNA Reveals the Usage of Multiple KSHV Genome Regions for Latent Replication
Source: PLoS Pathog. 2011 Nov 3;7(11):e1002365. doi: 10.1371/journal.ppat.1002365 (PMC3207954; doi:10.1371/journal.ppat.1002365)

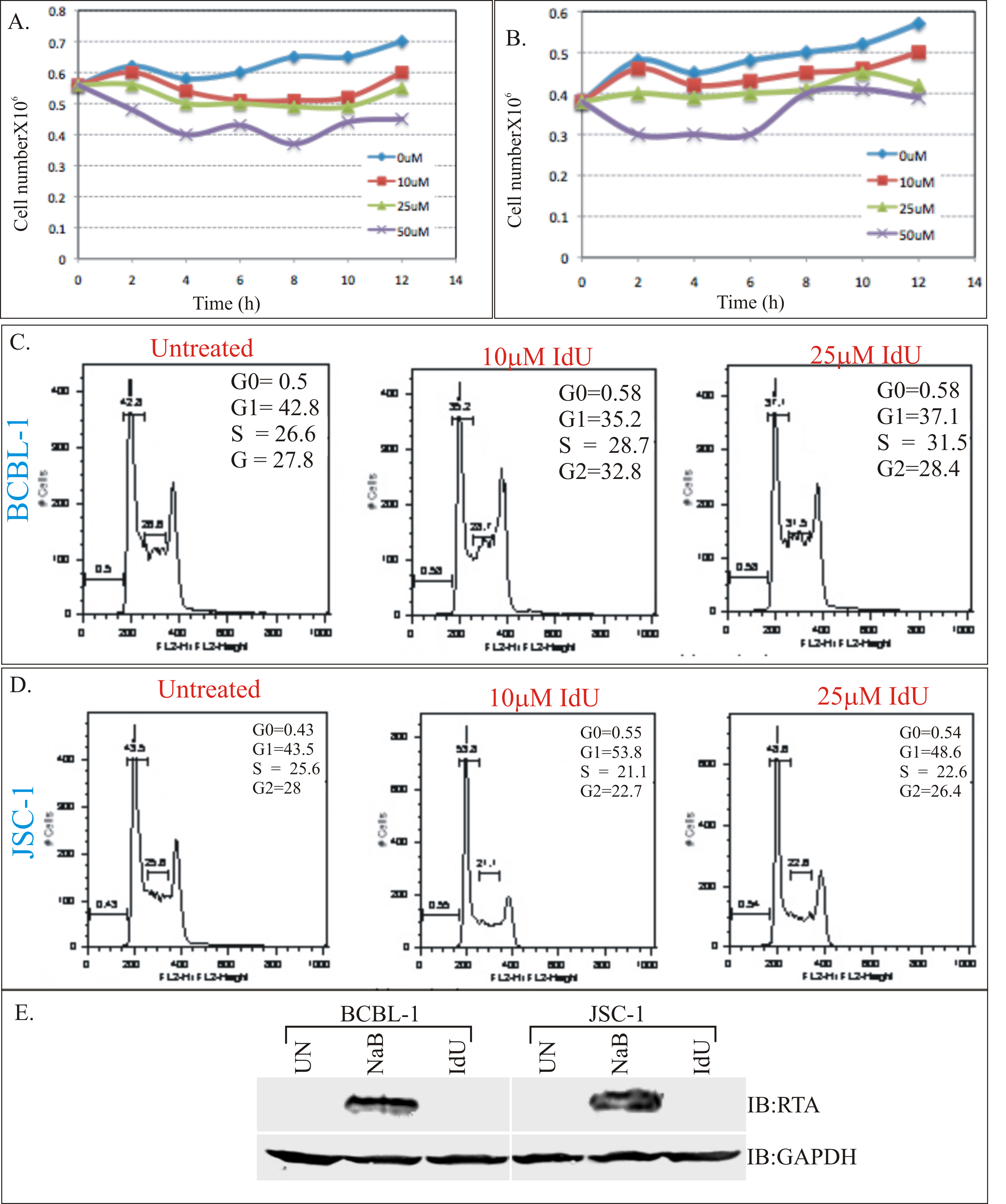

Supplement: Figure S1 — Halogenated nucleotide analog did not affect the growth patterns of KSHV infected Cells. A: Growth kinetics of BCBL-1 cells with various concentrations of IdU. B: Growth kinetics of JSC-1 cells with various IdU concentrations. C and D: Cell cycle profile of BCBL-1 and JSC-1 cells, respectively, with (10 and 25 uM) and without (Untreated) IdU pulsing. Pulsing with the nucleoside analog (IdU) did not significantly affect the growth of growth of the PEL cells (BCBL-1 and JSC-1). E. BCBL-1 and JSC-1 cells were treated with either 1 mM sodium butyrate (Histone Deacetylase inhibitor used for induction of lytic cycle) or 30 uM of IdU (halogenated nucleotide analog used for labeling) for 24 followed by detection of immediate early protein, RTA (replication and transcriptional activator) in a western blot assay. Levels of RTA were compared in un-induced (UN), Sodium butyrate (NaB) and in IdU treated (IdU) cells. IdU treatment did not show detectable levels of RTA. Anti-GAPDH antibody was used to show that the levels of total proteins were similar in all three samples. (TIF) [file ppat.1002365.s001.tif]

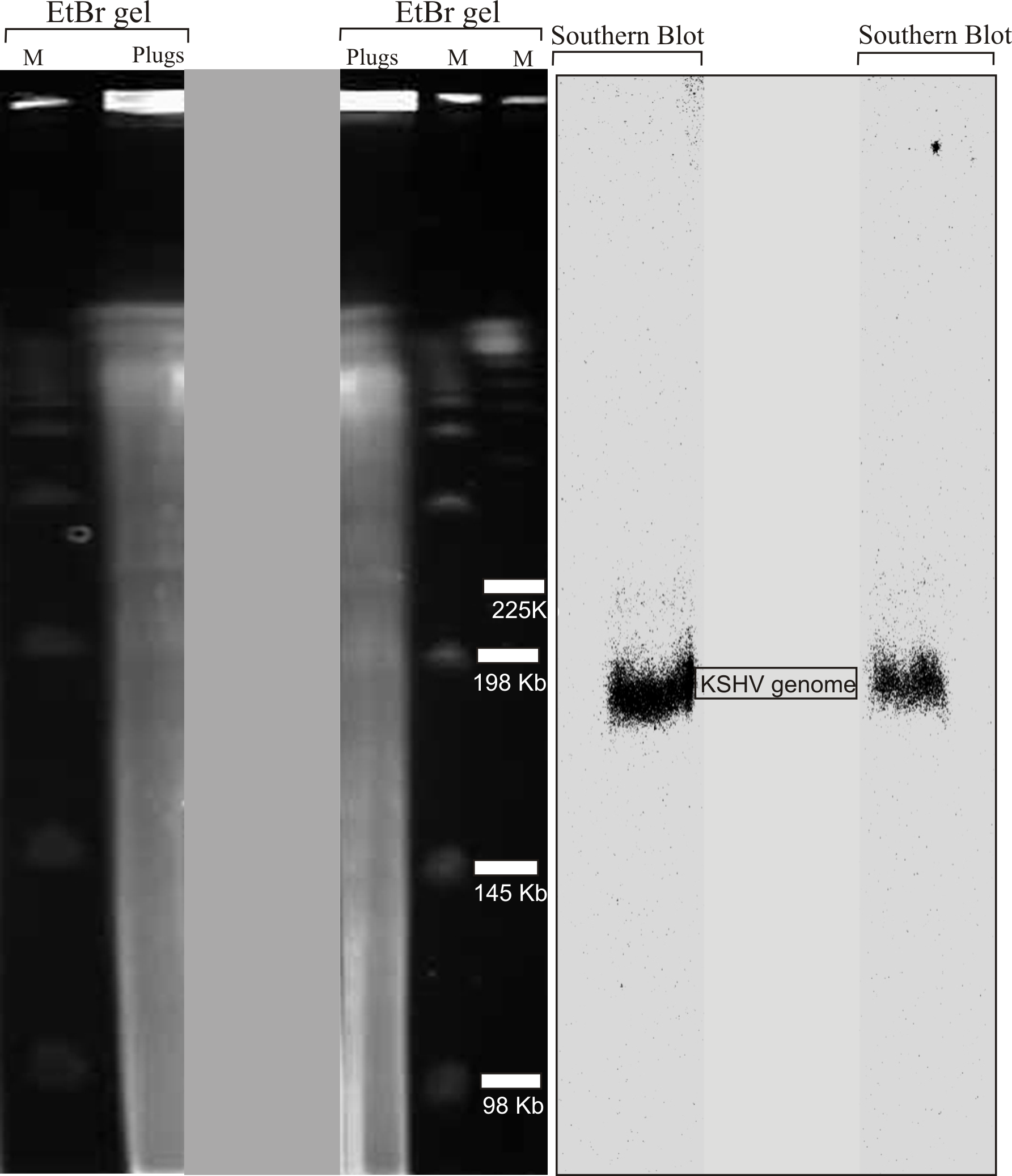

Supplement: Figure S2 — Pulse Field Gel Electrophoresis (PFGE) to isolate linear KSHV genomes. Agarose plugs containing labeled cells (IdU and CldU) were digested with PmeI to linearize the genome. These plugs were loaded onto a low melt agarose gel along with the pulse field DNA marker (M on both side of the plugs) and resolved by a pulse field gel electrophoresis. Gel containing the central portion of plugs (grey part of EtBr gel) was excised and stored for KSHV genome extraction. Both the sides of the gel (labeled as EtBr gel) were Sothern transferred and probed to detect the KSHV genome. Linear KSHV genome signals detected (165 kb signals on Southern blot) were aligned with the central portion of the gel to excise the KSHV genome by gelase treatment. (TIF) [file ppat.1002365.s002.tif]

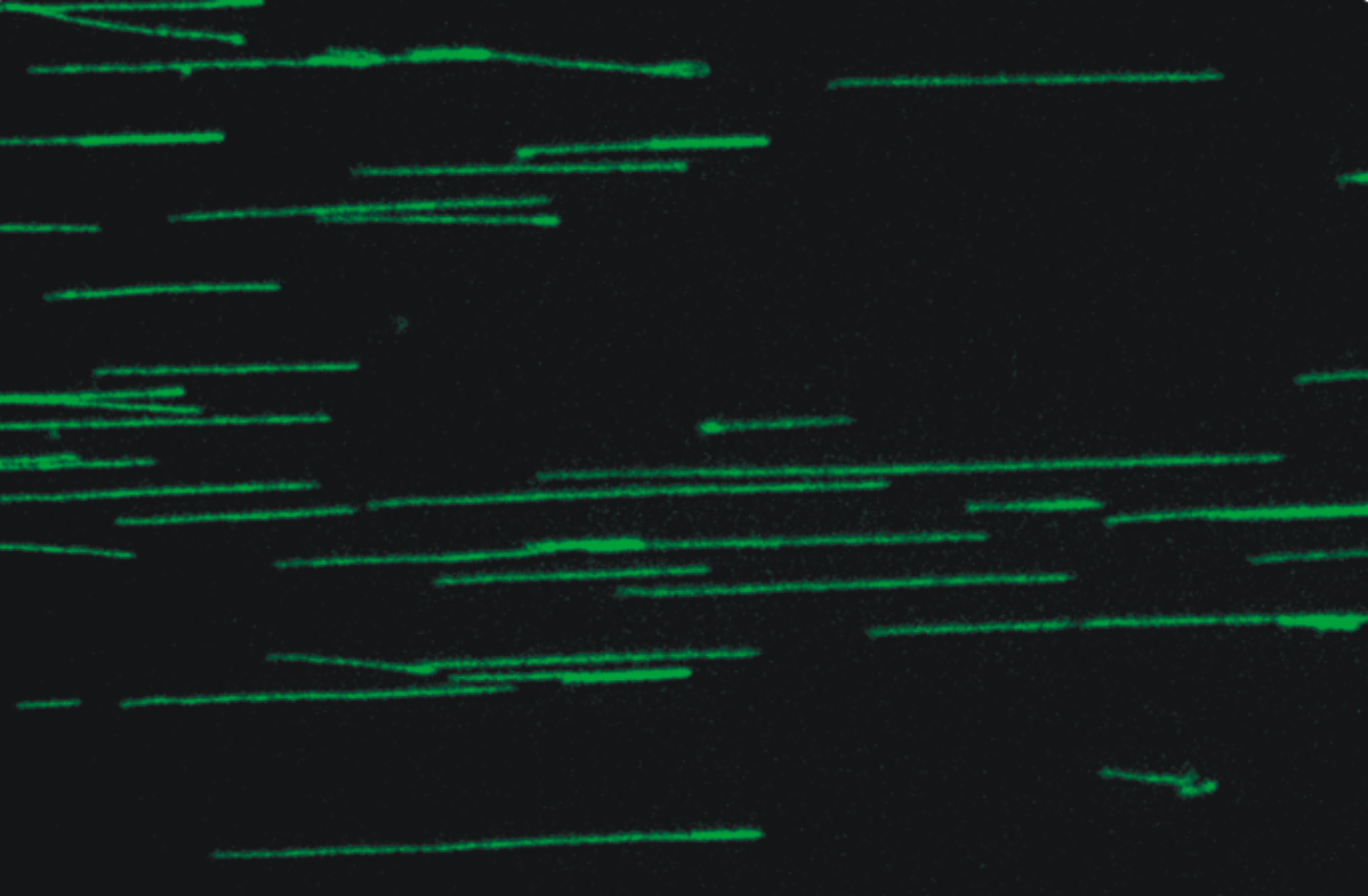

Supplement: Figure S3 — Visualization of stretched DNA on 3-aminopropyl-tri-ethoxysilane coated slides. BCBL-1 DNA extracted by the pulse field gel electrophoresis was stained with YOYO-1 dye to visualize after stretching the DNA by capillary force. The stretched DNA was imaged at 60X oil objective using Zeiss fluorescent microscope. (TIF) [file ppat.1002365.s003.tif]

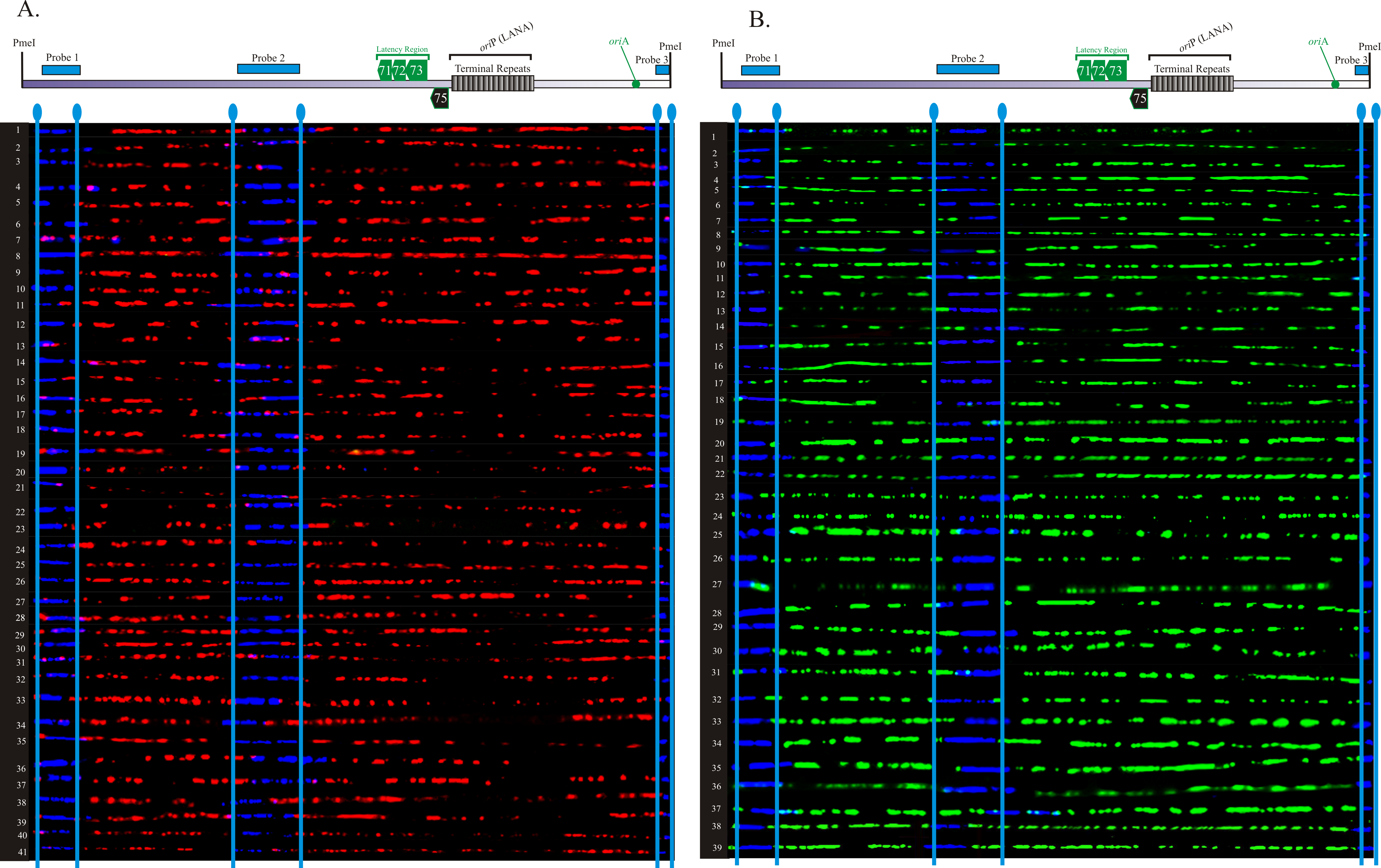

Supplement: Figure S4 — Fully substituted BCBL-1 KSHV genome. KSHV genome molecules replicated in BCBL-1 cells labeled with either the first label (A, Red) or the second label (B, Green). We collected forty one fully red and thirty nine fully green molecules while imaging molecules labeled with both the analogs. These molecules were used in the calculation of replication fork meovement. (TIF) [file ppat.1002365.s004.tif]

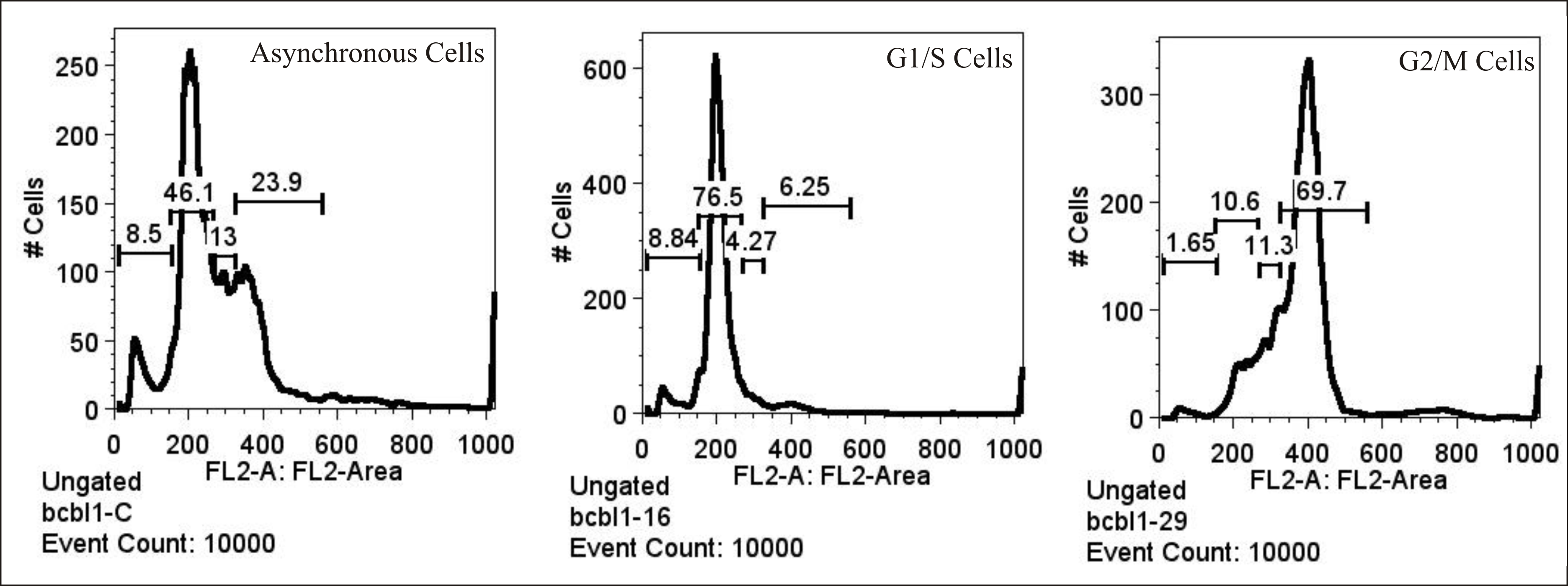

Supplement: Figure S5 — Cell cycle profiles of the elutriated BCBL-1 cells used in ChIP assay. Fraction 16 (BCBL1-16) which constituted over 80% cells in G1/S phase and fraction 29 (BCBL1-29) with over 80% cells in G2/M phase were used in ChIP assay. (TIF) [file ppat.1002365.s005.tif]

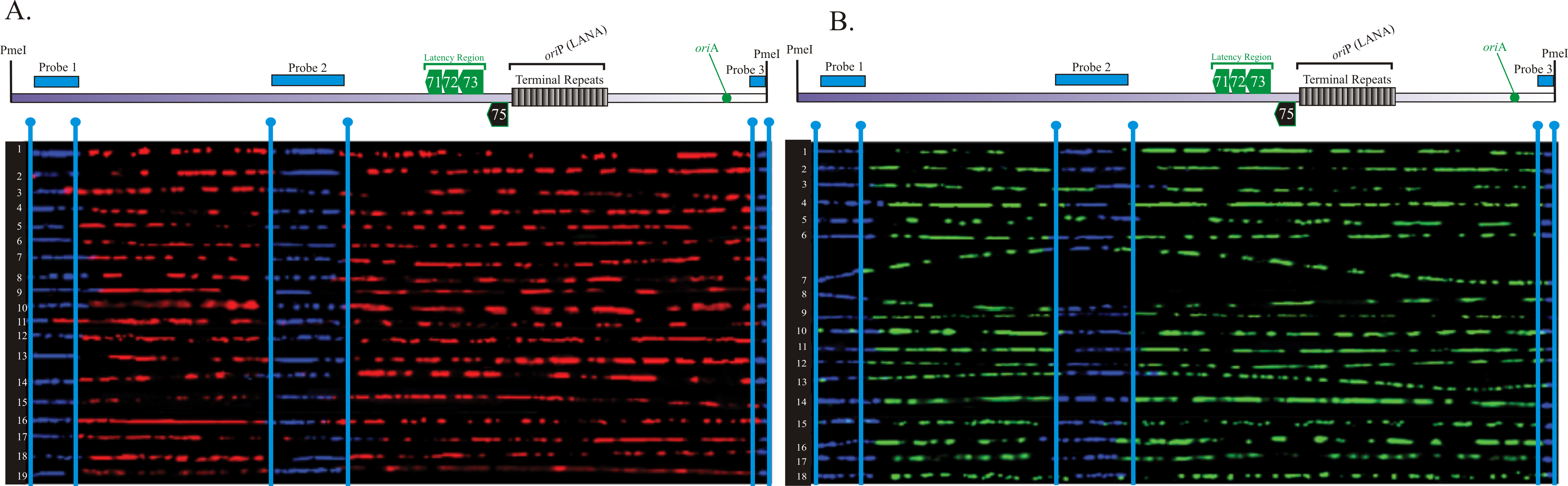

Supplement: Figure S6 — Fully substituted JSC-1 KSHV genome. KSHV genome molecules replicated in JSC-1 cells labeled with either the first label (A, Red) or the second label (B, Green). There were images of nineteen fully red and eighteen fully green molecules in JSC-1 cells. (TIF) [file ppat.1002365.s006.tif]
